# Latent tuberculosis coinfection in mild COVID-19 is associated with a distinct immune cell phenotype marked by enhanced cytotoxic degranulation and mitochondrial alterations

**DOI:** 10.3389/fimmu.2025.1566449

**Published:** 2025-05-19

**Authors:** Carlos Peña-Bates, Lucero A. Ramón-Luing, Julio Flores-Gonzalez, Enrique Espinosa, María F. Martinez-Moreno, Karen Medina-Quero, Marco A. Vargas-Hernandez, Norma A. Téllez-Navarrete, Fernando M. Sosa-Gomez, Eduardo Becerril-Vargas, Miguel Ángel Salazar, Leslie Chavez-Galan

**Affiliations:** ^1^ Laboratory of Integrative Immunology, Instituto Nacional de Enfermedades Respiratorias Ismael Cosío Villegas, Mexico City, Mexico; ^2^ Laboratory of Immunology, Escuela Militar de Graduados de Sanidad, Universidad del Ejército y Fuerza Aérea Mexicanos, Mexico City, Mexico; ^3^ Department of Healthcare Coordination, Instituto Nacional de Enfermedades Respiratorias Ismael Cosío Villegas, Mexico City, Mexico; ^4^ Department of Occupational and Preventive Health, Instituto Nacional de Enfermedades Respiratorias Ismael Cosío Villegas, Mexico City, Mexico; ^5^ Laboratory of Clinical Microbiology, Instituto Nacional de Enfermedades Respiratorias Ismael Cosío Villegas, Mexico City, Mexico; ^6^ Tuberculosis Clinic, Instituto Nacional de Enfermedades Respiratorias Ismael Cosío Villegas, Mexico City, Mexico

**Keywords:** latent tuberculosis, COVID-19, coinfection, T lymphocytes, mitochondrial changes

## Abstract

**Introduction:**

The chronic nature of latent tuberculosis infection (LTBI) allows it to coexist with diverse pathologies. However, it remains unclear whether immune alterations associated with LTBI influence COVID-19 coinfection and patient outcomes. This study aims to compare the immune phenotype of patients with LTBI/COVID-19 to those with COVID-19 alone, in order to assess whether latent tuberculosis infection induces significant immune cell alterations.

**Methods:**

Peripheral blood mononuclear cells were cultured and stimulated with the SARS-CoV-2 Spike protein and *Mycobacterium bovis* Bacillus Calmette-Guérin (*M. bovis* BCG) to evaluate cellular distribution and function.

**Results:**

the LTBI/COVID-19 group exhibited a narrower range of symptoms and required less complex treatment regimens than the COVID-19 group. The cellular evaluation revealed that individuals with COVID-19 displayed a distinct immune profile, characterized by a predominance of monocytes expressing pro-inflammatory and regulatory markers, including TNFR2, HLA-DR+TNFR2, and CD71. While CD4+ T cell subpopulation distribution and function were similar across groups, LTBI/COVID-19 and COVID-19 exhibited similar frequencies of CD8+perforin+ and CD8+Granzime B+ T cells. However, LTBI/COVID-19 displays lower soluble levels of granzyme B and perforin in culture supernatants and perforin, granulysin, and sFas in plasma compared to COVID-19. Notably, CD8+ T cells from LTBI/COVID-19 showed higher antigen-specific degranulation than COVID-19. Moreover, LTBI/COVID-19 individuals predominantly displayed CD4+ and CD8+ T cells with highly polarized, compact mitochondria at baseline, which remained unchanged under stimulation. In contrast, COVID-19 had T cells with highly polarized, fragmented mitochondria at baseline, a profile that persisted under stimulation.

**Conclusion:**

The findings reveal significant alterations in monocytes and T cells of individuals with LTBI/COVID-19, suggesting that co-infection may induce changes in the cellular phenotype and cytotoxic function of CD8 T cells.

## 1 Introduction

The World Health Organization (WHO) reported that tuberculosis (TB) caused 1.25 million deaths worldwide in 2023 and noted 10.8 million active TB cases (1). Those with active TB typically have a cough lasting three weeks or more, and they can spread the infection (2). Most people exposed to the bacteria *Mycobacterium tuberculosis* (Mtb) have an immune response that controls but does not eliminate it, leading to latent TB infection (LTBI). Individuals with LTBI show no symptoms and cannot spread the disease; although not a source of infection, they are considered a reservoir for future active TB cases. At least one-fourth of the world’s population has LTBI, making it a significant global health issue (3).

An individual with LTBI can remain in this state throughout his or her life without developing TB. Although it may coexist with various comorbidities and infections, these are at high risk for developing pulmonary TB (4, 5). It has been reported that CD4+ T cell depletion associated with severe COVID-19 and corticosteroid-based treatments could induce the activation of pulmonary TB in individuals with LTBI (6, 7). However, there is very little data on the immunological consequences of LTBI in co-infection with COVID-19 (8).

The SARS-CoV-2 infection, the causative agent of COVID-19, induces a wide clinical spectrum ranging from asymptomatic to severe disease (9). Patients who develop respiratory complications and severe disease exhibit significant immune dysregulation, including cytokine release syndrome, exhausted NK and CD8+ T cells, mitochondrial metabolic alterations in T cells, and decreased secretion of type I interferons (9, 10). Collectively, these abnormalities often result in fatal outcomes.

The introduction of SARS-CoV-2 vaccines significantly reduced the number of individuals with severe disease and associated mortality rates (11). Experimental studies have demonstrated that SARS-CoV-2 vaccines, including AZD1222/Covishield and BV152/Covaxin, elicit a memory response in CD4+ and CD8+ T cells that persists over time, providing long-term protection through T cell-induced immune pathways (11–13). A murine model has proposed that prior exposure to M. tuberculosis (Mtb) may confer protection against SARS-CoV-2; similarly, it has been suggested that vaccination with Bacillus Calmette-Guérin (BCG) might provide comparable protection (14–16). However, the results of these studies remain controversial and vary depending on the population and the type of vaccine administered during early childhood (17).

Data indicate that aging patients with LTBI/COVID-19 coinfection exhibit modulation of their immune response, which has been associated with the presence of Mtb infection (18, 19). Conversely, another study found that patients infected with Mtb (whether LTBI or active TB) have a limited SARS-CoV-2-specific response, notably reduced IFN-γ release, suggesting a potential detriment to an adequate immune response during LTBI/COVID-19 coinfection (20).

Additionally, studies have examined the impact of SARS-CoV-2 vaccination on individuals with active TB who have received SARS-CoV-2 vaccination. These cells were exposed to SARS-CoV-2 antigens, and notable immunological alterations were identified, including a reduction in CD8+CD69+ and CD8+TNF+ T cells and an increase in CD4+IL-10+ T cells, suggesting that Mtb infection promotes an anti-inflammatory profile (21).

Despite efforts to elucidate the impact of LTBI during acute COVID-19, several questions about Mtb and SARS-CoV-2 coinfection remain unanswered. This study analyzes the immunologic phenotype during mild COVID-19 in a group of individuals who have received a mixture of SARS-CoV-2 vaccines, were previously vaccinated with BCG in early childhood, and have been identified as having latent tuberculosis infection (LTBI) to assess its influence on patient outcomes.

## 2 Materials and methods

### 2.1 Ethics statement

This study was approved by the Institutional Ethics Committee of the Instituto Nacional de Enfermedades Respiratorias Ismael Cosio Villegas (INER, protocol number B23–23). All participants signed a written informed consent. All procedures in this work followed the ethical standards indicated in the Helsinki Declaration.

### 2.2 Study populations

From October 2023 to May 2024, healthcare workers from the Instituto Nacional de Enfermedades Respiratorias Ismael Cosio Villegas (INER) were invited to participate in a study. The medical staff evaluated all participants in the Occupational and Preventive Health Department for clinical evaluation. 110 healthcare workers (over 18 years) were recruited.

All individuals have received the SARS-CoV-2 vaccination, and 94% received the BCG vaccination in early childhood. Eight subjects were excluded from the study due to their decision to discontinue participation or insufficient sample size. Furthermore, subjects who had been diagnosed with Human Immunodeficiency Virus (HIV) infection, active TB, cancer, chronic obstructive pulmonary disease, solid organ transplant recipients, or those who had been prescribed immunosuppressive or anticoagulant therapy were excluded from the study.

**Figure 1:**
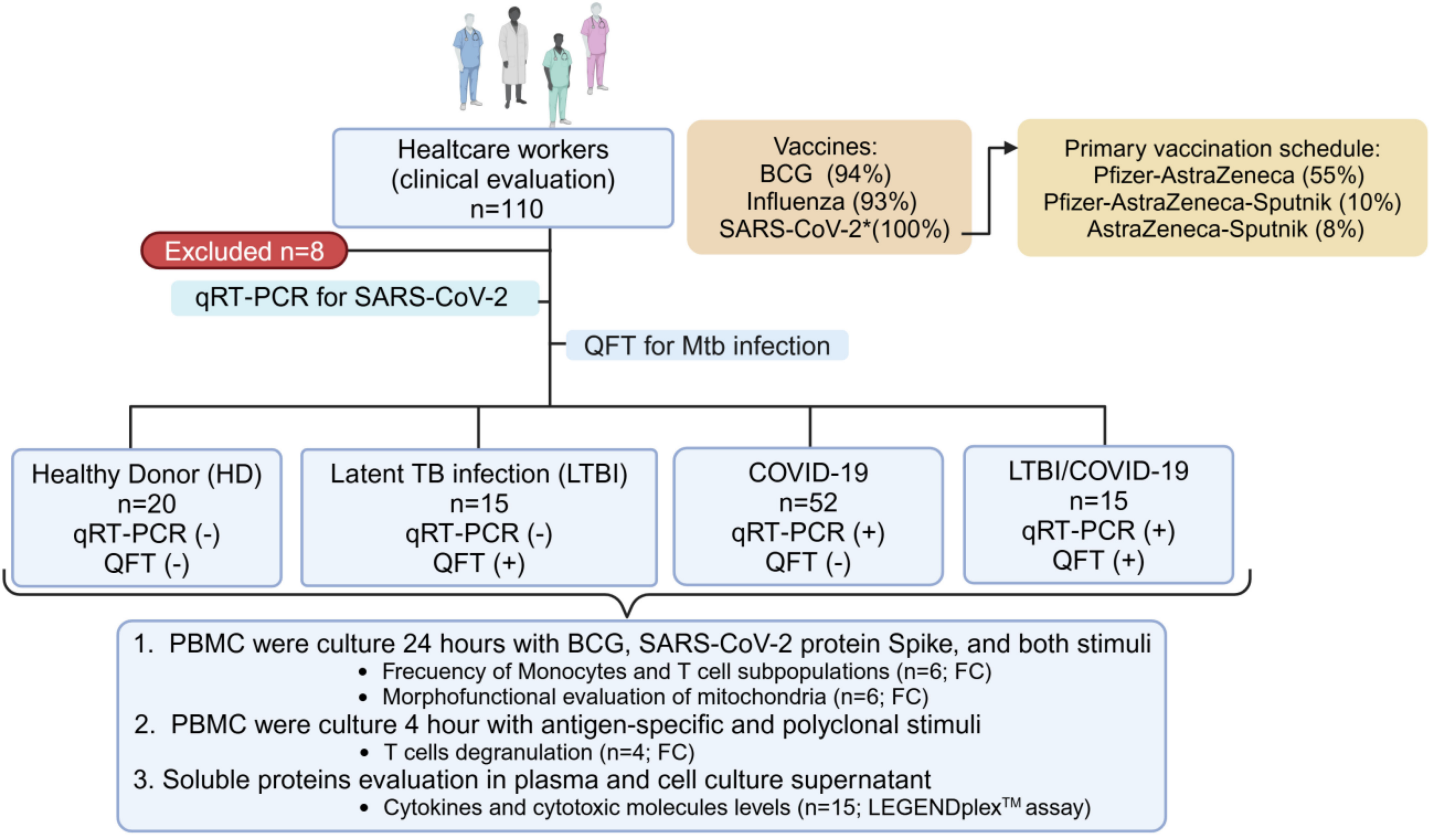
Workflow of recruited individuals. 110 healthcare workers (over 18 years) were recruited. All have received SARS-CoV-2 vaccination. Eight subjects were excluded from the study due to their decision to discontinue participation or insufficient sample size, and 102 were classified according to the reverse transcriptase polymerase chain reaction (qRT‐PCR) and QuantiFERON-Gold plus (QFT) tests, to identify the SARS-CoV-2 or *Mycobacterium tuberculosis* (Mtb) infection, respectively. The study population was divided into four groups: healthy individuals (HD, n=20), individuals with latent TB infection (LTBI, n=15), individuals with confirmed mild SARS-CoV-2 infection (COVID-19, n=52), and individuals with latent TB infection and confirmed mild SARS-CoV-2 infection (LTBI/COVID-19, n=15). The cells of these individuals were evaluated to measure monocyte and T cell subpopulations’ parameters (n=6 per group), determine the degranulation capacity of CD8+ and CD4+ T cells (n=4 per group), and assess cytokines in plasma (n=15 per group) and culture supernatant (n=6 per group) under specific stimuli. FC, Flow Cytometry; PBMC, peripheral blood mononuclear cells. Created in https://BioRender.com.

A reverse transcriptase polymerase chain reaction for SARS-CoV-2 (qRT-PCR, BioFire Diagnostics, LLC, USA) and QuantiFERON-Gold plus (QFT, Qiagen, HD, Germany) to identify Mtb infection were performed in all participants. They were classified into four groups: healthy donors (HD, n=20), latent TB infection (LTBI, n=15), mild COVID-19 (COVID-19, n=52), and coinfection LTBI/mild COVID-19 (LTBI/COVID-19, n=15) (details in **Figure 1**). This study only included mild COVID-19 patients, and according to the WHO definition and the institutional clinical staff, mild COVID-19 is considered when patients have no viral pneumonia and hypoxemia and present an oxygen saturation >92%; consequently, no patient required hospitalization or supplemental oxygen. Additionally, showed at least 2 of the following symptoms: cough, fever, or headache, accompanied by at least 1 of the following signs or symptoms: difficulty breathing, anosmia, joint or muscle pain, throat pain, and nasal congestion (22, 23).

### 2.3 Peripheral blood mononuclear cells

20 ml of EDTA blood samples were collected from healthcare workers into BD Vacutainer tubes (BD Biosciences, CA, USA). Then, peripheral blood mononuclear cells (PBMC) were isolated by a density gradient (Lymphoprep™, Accurate Chemical-Scientific, NY, USA). The trypan blue (Gibco™, NY, USA) exclusion assay was used to determine the number of viable cells, and a minimum of 90% viability was required to process the use of the cells. Plasma was obtained and stored at −70 °C until use. To note, cells used for the *in vitro* studies were selected from those patients that did not present comorbidities to avoid variables.

### 2.4 Culture of BCG and *in vitro* infection assay

A suspension of BCG Pasteur strain 1172 P2 was prepared in the Middlebrook 7H9 broth medium BD (Becton Dickinson, USA) supplemented with oleic albumin dextrose catalase (OADC) (24). After a 21-day incubation period at 37°C, the mycobacterial stock solution was harvested. Aliquots of disaggregated mycobacterial stock cultures were prepared and stored at −70°C until required for *in vitro* infections. The mean concentration of the frozen BCG stock suspensions after disruption of mycobacterial clumps was determined by counting colony-forming units (CFU) on 7H10 agar plates in triplicate serial dilutions of the disaggregated stock suspensions.

For infection assay, a suspension of bacteria was prepared; briefly, an aliquot of bacteria was thawed and centrifuged at 6000 x g for 5 min. The bacterial pellet was resuspended in RPMI 1640 medium supplemented with 2 mM L-glutamine,1M HEPES (Gibco™, NY, USA), and 10% fetal bovine serum (Gibco™) and shaken in the presence of sterile 3 mm glass beads. The resulting mycobacterial suspension was centrifuged to remove residual large lumps, and single-cell suspensions of bacteria were used for the infection of PBMC. Then, PBMC (1×10^6^/mL) were infected with BCG Pasteur strain at a multiplicity of infection (MOI) of 1 (1 cell per 1 bacteria). The infected PBMC were incubated at 37°C for 4 hours, and no phagocytosed bacteria were eliminated by washing. Then, cells were stimulated, as described below.

### 2.5 PBMC stimulation

1×10^6^ of PBMC/mL of HD (n=6), LTBI (n=6), COVID-19 (n=6), and LTBI/COVID-19 (n=6) were plated in RPMI-1640 medium supplemented with 2 mM L-glutamine,1M HEPES (Gibco™), and 10% fetal bovine serum (Gibco™).

Three different stimuli conditions were performed: a) PBMC infected with BCG Pasteur strain (MOI 1), b) PBMC stimulated with the recombinant SARS-CoV-2 Spike (S1+S2) protein, here after called as only spike (Biolegend, CA, USA) at 1 μg/mL concentration and, c) PBMC were infected with BCG (MOI 1) plus the spike (1 μg/mL). The culture was maintained for 24 hours (h) at 37°C in a 5% CO_2_ humidified atmosphere. After the incubation, the supernatants were recovered and frozen at -20°C. The recovered cells were prepared for flow cytometry staining. Unstimulated PBMC (mock control) were cultured in the same conditions. Supernatant cultures were recovered and stored at -20°C until use.

### 2.6 Flow cytometry: staining and analysis strategy

1x10^6^ PBMC of HD (n=6), LTBI (n=6), COVID-19 (n=6), and LTBI/COVID-19 (n=6) were recovered at the end of the culture, and extracellularly stained with monoclonal antibodies (mAb) against CD2, CD3, CD14, CD16, HLA-DR, CD120a, CD120b, CD71, CD274, CD8, CD4, CD69, CD98, CD279, TIM3 (Biolegend, CA, USA), for 30 min at 4°C in the dark, washed with cell staining buffer (BioLegend) and fixed.

For intracellular staining, a mAb cocktail against T-bet, GATA-3, perforin, and granzyme B was used. Briefly, after extracellular staining, cells were washed with cell staining buffer and permeabilized with BD Cytofix/Cytoperm™ buffer (BD Biosciences) for 20 min at 4°C. Following, it was incubated with the intracellular mAb cocktail for 30 min at 4°C in the dark. Finally, the cells were washed and acquired.

For mitochondria evaluation, briefly, PBMC were stained for 15 min 37°C with mitotracker green FM (500 nM) and mitotracker Deep Red (250 nM) probes. Following, cells were washed, and an extracellular stain was done. MDIVI1 (Sigma-Aldrich, USA), a mitochondrial fusion inductor, allowed us to distinguish compacted from fractionated mitochondria, and FCCP (Sigma-Aldrich), an uncoupler of oxidative phosphorylation, allows us to obtain a polarized/depolarized mitochondrial mass control, were used as controls.

Data were acquired using a LSRFortessa™ BD flow cytometry with FACSDiva 6.1.3 software (BD Biosciences). Fluorescence Minus One (FMO) condition was stained and acquired in parallel to identify background levels of staining, and dead cells were excluded using the viability staining Zombie Red Dye solution (BioLegend). In each condition, at least 50,000 events per sample were acquired. The flow cytometry (FCS) data file was analyzed using Flow Jo (Flow Jo) ™ v10.10.1 (Flow Jo, LLC, OR, USA). More details of the antibodies used can be found in **Supplementary Table S1**.

The analysis strategy involves selecting live cells employing the viability plot (Zombie Red negative). Subsequently, the limitation of the single T cells through forward scatter (FSC-A vs. FSC-H) was performed. Subsequent PBMC were chosen through side scatter and forward scatter (FSC-A versus SSC-A), and a second singlets events plot was made (SSC-A versus SSC-H) (**Supplementary Figure S1A**).

Subsequently, PBMC was selected, and further analysis was conducted on CD2- or CD2+ cells, monocytes (CD2-CD14+), and T cells (CD2+CD3+), along with their CD4+ or CD8+ subpopulations. For monocytes, activation markers such as HLA-DR and CD71, as well as death or survival receptors TNFR1 and TNFR2, respectively, were evaluated. Moreover, classical monocytes (CD14+CD16-) and non-classical monocytes (CD14+CD16+) were assessed. In the T cell gate, activation (CD69) and exhausted-like phenotype (PD-1, and TIM-3) markers were assessed. In addition, into the T cells CD4+ gate, GATA-3 and T-BET were evaluated to define the Th1 or Th2 profile, while at the CD8+ gate, granzyme and perforin were analyzed to assess the presence of cytotoxic molecules (**Supplementary Figure S1A**).

The CD4+ and CD8+ gates were evaluated, utilizing MDVI1 and FCCP controls to delimit the cutoffs for mitochondrial fragmentation and polarization. Employing Mitotracker Green FM (a mitochondrial mass indicator) and Mitotracker Deep Red (a mitochondrial membrane potential-dependent indicator), cells were divided according to the presence of fractionated or compacted mitochondria and high or low polarized (**Supplementary Figure S1B**).

### 2.7 CD8+ T cell degranulation

For the polyclonal stimuli, 1×10^6^ PBMC/mL of HD (n=4), LTBI (n=4), COVID-19 (n=4), and LTBI/COVID-19 (n=4) were plated in RPMI-1640 medium supplemented and maintained at 37°C. A mAb anti-CD107a (Biolegend) (5 µL/mL) was added, and after 30 minutes, a mixture of phorbol-12-myristate-13-acetate with ionomycin (PMA/IO, Thermo Fisher Scientific, CA, USA) at 1X of was added. The cultures were incubated for 4 h, but 2 h before the end of the culture, monensin (0.002 mM, Biolegend) was added to the cell culture. Kinetics of CD8+ T cell degranulation was performed, including four time points: basal, 1, 2, and 4 h.

For antigen-specific stimuli, 1×10^6^ PBMC/mL of HD (n=4), LTBI (n=4), COVID-19 (n=4), and LTBI/COVID-19 (n=4) were plated in RPMI-1640 supplemented. Briefly, after 30 min of culture assay, anti-CD107a was added and stimulated with the spike and BCG for 4 h at 37°C. Two hours before the end of the culture assay, monensin was added. After polyclonal or antigen-specific stimuli, extracellular staining was done to identify CD3, CD4, and CD8.

Data were acquired using a FACSCanto II™ flow cytometer with FACSDiva 6.1.3 software (BD Biosciences). In each condition, at least 50,000 events per sample were acquired. The flow cytometry (FCS) data file was analyzed using Flow Jo (Flow Jo) ™ v10.10.1 (Flow Jo, LLC, OR, USA).

### 2.8 Soluble molecules evaluation

Following instructions provided by the manufacturer (BioLegend), IL-2, IL-4, IL-6, IL-10, IL-17A, IFN-γ, TNF-α, soluble Fas, soluble FasL, Granzyme A, Granzyme B, Perforin, and Granulysin were measured in plasma samples and supernatants from culture assays using the LEGENDplex human CD8/NK panel (kit’ details in **Supplementary Table S1**). Data were collected using a FACSAccuri C6™ with CFlow software (BD Biosciences).

### 2.9 Statistical analysis

The D’Agostino-Pearson test was used to test the normality of data. Non-normally distributed variables are shown as median value and interquartile range (IQR, 25–75). Kruskal–Wallis test with Dunnett’s post-test was used for multiple comparisons. In relation to the clinical laboratory data, they presented a normal distribution, so they were analyzed by means of the One-Way Anova test, the data were expressed as the mean and the respective maximum and minimum values. p<0.05 were considered statistically significant (GraphPad Software, V9.0.2). Details on size sample are provided in the **Supplementary Material**.

The Heat map was created in the interface of Morpheus, in which a matrix of values is mapped to a matrix of colors. The values are assigned to colors using the minimum and maximum of each row independently. Versatile matrix visualization and analysis software (Morpheus, https://software.broadinstitute.org/morpheus).

## 3 Results

### 3.1 Demographic and clinical characteristics

Demographic characteristics were compared between groups (**Supplementary Table S2**). In summary, the cohort consisted of young adults, predominantly female, who had received at least two doses of the SARS-CoV-2 vaccine. Most patients received a combination of vaccines, with the following combinations standing out: Pfizer-AstraZeneca (55%), Pfizer-AstraZeneca-Sputnik (10%), and AstraZeneca-Sputnik (8%). Approximately 70% received their last dose in December 2022, 20% in February 2022, and 10% received their last vaccine in November 2021. All subjects received the last doses of the vaccine in similar data before being enrolled.

Overweight and obese were the most common comorbidities in the COVID-19 and LTBI/COVID-19 groups, respectively. 49% reported contact with TB patients (because of the nature of their work). With respect to SARS-CoV-2 exposure, the majority of individuals had been ill and diagnosed at least once with COVID-19, although in the HD and LTBI groups, it was not possible to know the time of their last SARS-CoV-2 infection (**Supplementary Table S2**).

No significant differences between the groups regarding hematological and biochemical parameters (**Supplementary Table S3**). In contrast, LTBI/COVID-19 exhibited a narrower range of symptoms compared to COVID-19; symptoms such as asthenia, adynamia, abdominal pain, fatigue, dyspnea, ageusia, and anosmia were not reported (**Supplementary Figure S2**, upper panel). As a result, treatment schedules differed between the groups: 73% of LTBI/COVID-19 cases were managed adequately with paracetamol, levodropropizine, and fexofenadine, whereas the COVID-19 group required more complex treatment regimens (**Supplementary Figure S2**, lower panel).

### 3.2 Circulating TNFR1+ monocytes are predominant in LTBI/COVID-19, whereas COVID-19 has TNFR2+ monocytes

**Figure 2:**
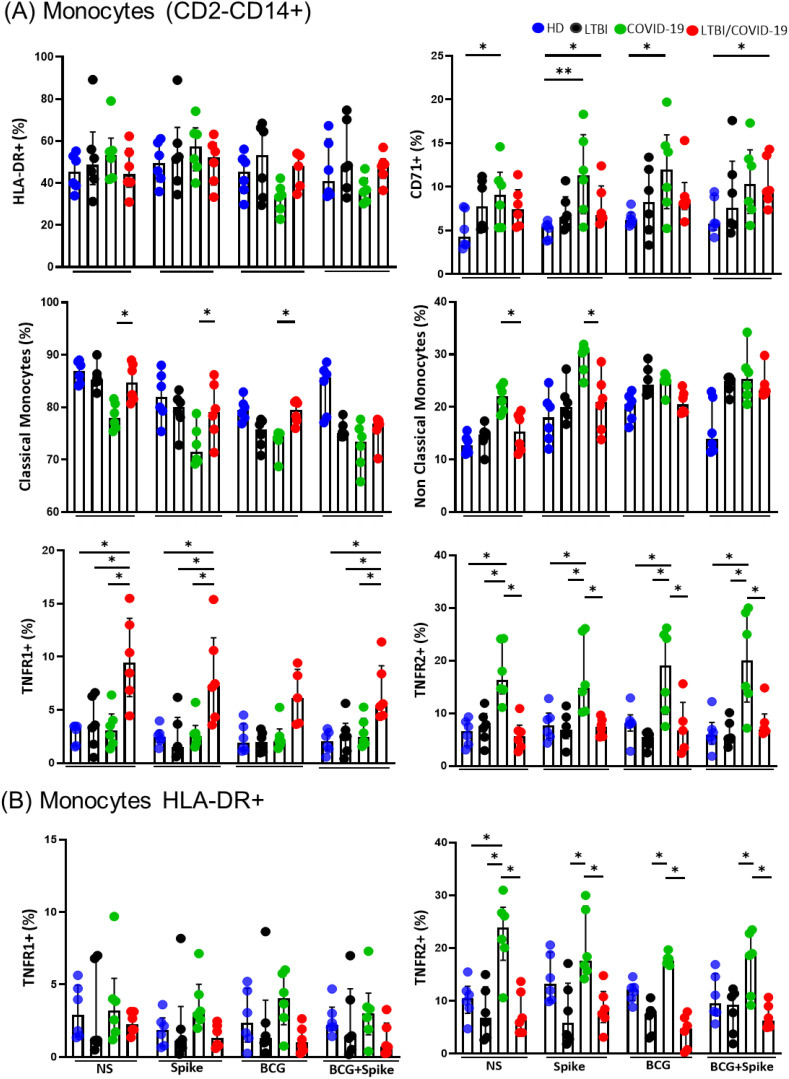
The expression of the receptor on monocytes is different in the LTBI/COVID-19 vs COVID-19 group. Peripheral blood mononuclear cells stimulated with BCG (MOI 1:1) and S protein (1μg/mL) were cultured for 24 hours, recovered, and evaluated by flow cytometry, n=6 per group. Into the gate CD2-CD14+ (monocytes), the frequency of monocytes HLA-DR+, classical monocytes CD14+, Non-Classical monocytes CD14+CD16+, CD71+, and TNFR1+ and TNFR2+ **(A)** was evaluated. Into the gate monocytes HLA-DR+, the frequency of TNFR1+ and TNFR2+ was evaluated **(B)**. Data are represented as medians with an interquartile range (IQR, 25-75), and each point represents individual data. The statistical comparison was performed using Kruskal-Wallis’s test, *p<0.05, **p<0.01. NS, not stimulated; HD, healthy donor; LTBI, latent tuberculosis infection; COVID-19, individual with COVID-19; LTBI/COVID-19, individual with latent tuberculosis and COVID-19 coinfection.

Within the monocyte gate (CD2-CD14+), the expression of HLA-DR was evaluated, and no significant differences were observed (**Figure 2A**, up). However, compared to HD, the frequency of monocytes expressing the transferrin receptor (CD71), an indirect activation marker, was increased in COVID-19 individuals without stimulation (p<0.05), in response to spike (p<0.01) and to BCG (p<0.05). Meanwhile, LTBI/COVID-19 showed an increase in the frequency of CD71+ monocytes in response to spike and BCG+spike (**Figure 2A**, up).

Subsequently, the frequency of classical (CD14+) and non-classical (CD14+CD16+) monocytes was evaluated. It is observed that COVID-19 shows a decreased frequency of classical monocytes compared to LTBI/COVID-19 (p<0.05), and it is maintained even with Spike and BCG stimulus (**Figure 2A**, mild). In contrast, COVID-19 has increased the frequency of non-classical monocytes compared to LTBI/COVID-19 (p<0.05), and it was observed without stimuli or with spike (**Figure 2A**, mild).

The expression of TNFRs was evaluated due to their involvement in COVID-19 severity (25, 26). Compared to HD and COVID-19, LTBI/COVID-19 had an increased frequency of TNFR1+ monocytes without stimulation (p<0.05), and this increase was sustained upon stimulation with spike (p<0.05) and BCG/spike (p<0.05). Conversely, compared to HD and LTBI/COVID-19, COVID-19 exhibited an increased frequency of TNFR2+ monocytes without stimulation (p<0.05), which persisted after stimulation with a spike (p<0.05), BCG (p<0.05), and BCG/spike (p<0.05) (**Figure 2A**, down).

Given that TNFR1 and TNFR2 mediate opposing functions, we investigated their expression specifically on antigen-presenting monocytes (HLA-DR+). We found that the frequency of HLA-DR+TNFR1+ monocytes was not significantly altered. However, at baseline, the frequency of HLA-DR+TNFR2+ monocytes was elevated in COVID-19 compared to HD, LTBI, and LTBI/COVID-19 (p<0.05). This increase persisted under stimulation in COVID-19 individuals compared to LTBI and LTBI/COVID-19 (p<0.05) (**Figure 2B**).

### 3.3 Activation and Th1/Th2 profile of CD4+ T cell is not modified during LTBI/COVID-19 coinfection

Data showed that the frequency of CD4+ and CD8+ T cells and the CD4/CD8 ratio were not different between groups (**Supplementary Figure S3**). Activation of T cells did not show important differences, only to note that HD increased the frequency of CD4+CD69+ T cells after BCG stimulation, whereas the frequency of CD8+CD69+ T cells was lower in HD at baseline compared to other groups (**Supplementary Figure S4**).

**Figure 3:**
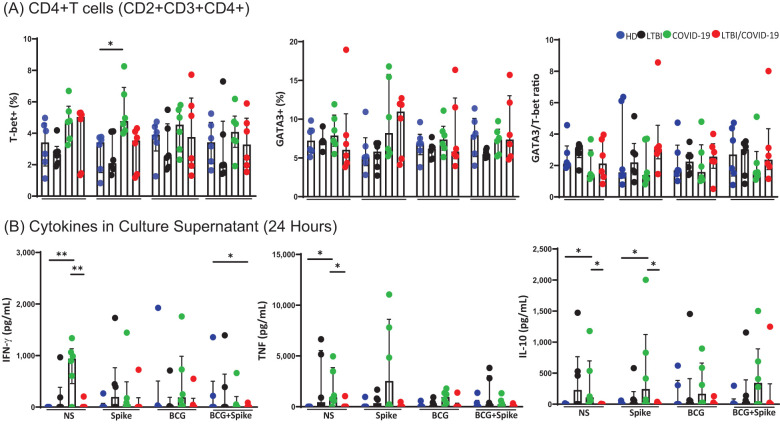
Th1 and Th2 responses are at comparable levels, with no evident predominance of one over the other. Peripheral blood mononuclear cells stimulated with BCG (MOI 1:1) and spike (1μg/mL) were cultured for 24 hours, recovered, and evaluated by flow cytometry, n=6 per group. Into the gate, CD2+CD3+ (T cells), the frequency of T CD4+ nuclear markers T-bet (Th1) or GATA-3 (Th2) **(A)**, and assessment of cytokines in culture supernatant by LEGENDplex™ **(B)** were evaluated. Data are represented as medians with an interquartile range (IQR, 25-75), and each point represents individual data. The statistical comparison was performed using Kruskal-Wallis’s test, *p<0.05, **p<0.01. NS, not stimulated; HD, healthy donor; LTBI, latent tuberculosis infection; COVID-19, individual with COVID-19; LTBI/COVID-19, individual with latent tuberculosis and COVID-19 coinfection.

Next, we evaluated whether CD4+ T cell function was altered by assessing Th1 (T-bet) and Th2 (GATA3) profiles. Despite COVID-19 individuals having a discreet increase in the frequency of CD4+T-bet+ T cells following spike stimulation, compared to HD, the GATA3/T-bet ratio was not modified (**Figure 3A**). We measured pro- and anti-inflammatory cytokines in the supernatant to confirm the inflammatory profile. At baseline, compared to HD and LTBI/COVID-19, COVID-19 had elevated levels of pro-inflammatory cytokines IFN-γ (p<0.01) and TNF (p<0.05). However, COVID-19 individuals also showed increased levels of the anti-inflammatory cytokine IL-10 at baseline (p<0.05) and after spike stimulation (p<0.05) (**Figure 3B**); all together suggested that the Th1/Th2 profile is not altered even under the coinfection context. Plasmatic levels of selected pro- and anti-inflammatory cytokines did not differ significantly between groups (**Supplementary Figure S5A**).

### 3.4 CD8+ T cells of LTBI/COVID-19 show similar behavior to those of COVID-19, but soluble cytotoxic molecules are decreased

**Figure 4:**
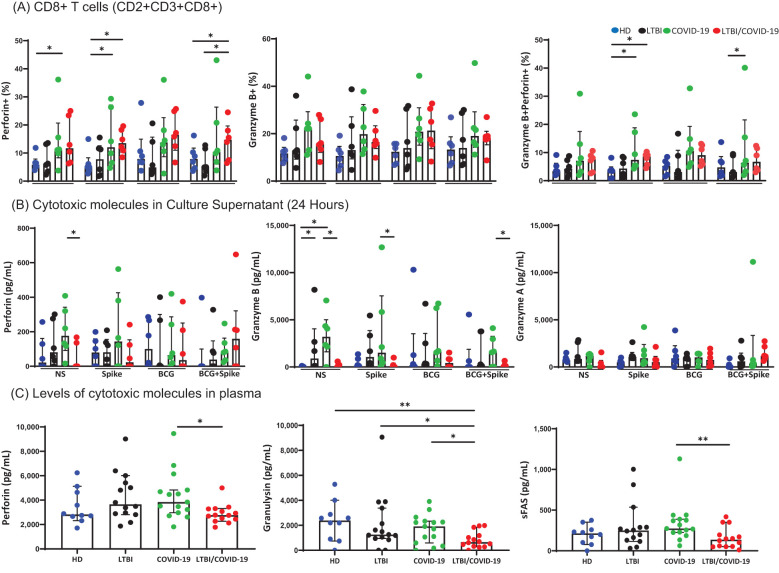
Secretion of cytotoxic molecules differ in LTBI/COVID-19 vs COVID-19 individuals. Peripheral blood mononuclear cells stimulated with BCG (MOI 1:1) and spike (1μg/mL) were cultured for 24 hours, recovered, and evaluated by flow cytometry. Frequency of CD8+Perforin+, CD8+ Granzyme B+, and CD8+ Granzyme B+Perforin+ T cells (CD2+CD3+ gate), n=6 per group **(A)**. Levels of cytotoxic molecules in the culture supernatant evaluated by LEGENDplex assay, n=6 per group **(B)**. Plasma levels of cytotoxic molecules were evaluated by LEGENDplex assay, n=10 (HD) and n=15 (LTBI, COVID-19, and LTBI/COVID-19) **(C)**. Data are represented as medians with an interquartile range (IQR, 25-75), and each point represents individual data. The statistical comparison was performed using Kruskal-Wallis’s test, *p<0.05, **p<0.01. NS, not stimulated. HD, healthy donor; LTBI, latent tuberculosis infection; COVID-19, individual with COVID-19; LTBI/COVID-19, individual with latent tuberculosis and COVID-19 coinfection.

Compared to HD, COVID-19 has increased the CD8+Perforin+ T cells’ frequency at baseline (p<0.05). In response to spike stimuli, both COVID-19 and LTBI/COVID-19 showed an increased frequency of T CD8+Perforin+ cells (p<0.05) compared to HD. Additionally, LTBI/COVID-19 maintained a high frequency when cells were stimulated with BCG/spike compared to HD and LTBI (p<0.05). The frequency of CD8+Granzyme B+ T cells did not differ significantly between groups or stimuli. However, the frequency of T CD8+ cells double-positive for Granzyme B and Perforin increased in both COVID-19 and LTBI/COVID-19 compared to HD upon spike stimulation (p<0.05), and COVID-19 maintained this high frequency with BCG/spike stimulation (p<0.05) (**Figure 4A**).

Following, soluble cytotoxic molecule levels were evaluated in the culture supernatant. Compared to COVID-19, LTBI/COVID-19 produced lower levels of perforin and granzyme B at baseline (p<0.05), a pattern that persisted after stimulation with spike and BCG/spike (p<0.05). Granzyme A levels did not differ between groups (**Figure 4B**). Furthermore, plasmatic levels of perforin and sFas (p<0.01), were lower in LTBI/COVID-19 compared to COVID-19 (**Figure 4C**). Granulysin levels were also lower in LTBI/COVID-19 compared to COVID-19, LTBI, and HD (p<0.05, p<0.01) (**Figure 4C**). However, systemic levels of granzyme A, granzyme B, and sFasL did not differ significantly between groups (**Supplementary Figure S5B**).

### 3.5 CD8+ T cells from LTBI/COVID-19 have not an exhausted-like phenotype and exhibit high antigen-specific degranulation capacity

**Figure 5:**
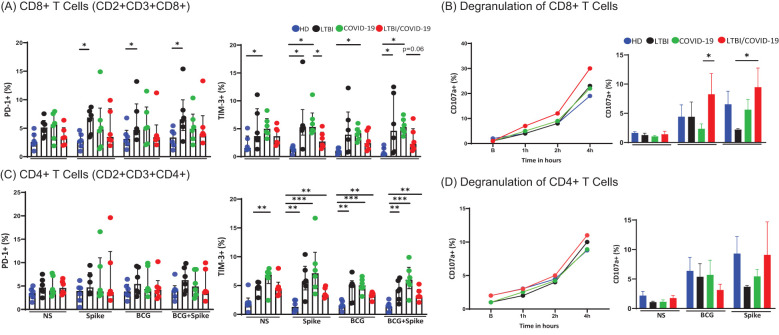
CD8+ T cell degranulation and cytotoxic regulation remain unaltered in LTBI/COVID-19 individuals. Peripheral blood mononuclear cells stimulated with BCG (MOI 1:1) and spike (1μg/mL) were cultured for 24 hours, recovered, and evaluated by flow cytometry (n=6 per group). Then, into the gate, CD2+CD3+ (T cells), the frequencies of CD8+PD-1+, CD8+TIM-3+ **(A)** and CD4+PD-1+, CD4+TIM-3+ were evaluated **(C)**. Peripheral blood mononuclear cells stimulated with PMA/IO (1X) as a polyclonal stimulus or BCG (MOI 1:1) and spike (1μg/mL) were cultured for 4 hours, recovered and evaluated by flow cytometry. The CD107a expression as a marker of degranulation (n=4 per group). Then, into the gate, CD2+CD3+ (T cells), the frequencies of CD8+CD107a+ **(B)**, and CD4+CD107a+ were evaluated **(D)**. Data are represented as medians with an interquartile range (IQR, 25-75), and each point represents individual data. The statistical comparison was performed using Kruskal-Wallis’s test, *p<0.05, **p<0.01, ***p<0.001. NS, not stimulated. HD, healthy donor; LTBI, latent tuberculosis infection; COVID-19, individual with COVID-19; LTBI/COVID-19, individual with latent tuberculosis and COVID-19 coinfection.

Given the discrepancy between intracellular and soluble cytotoxic molecules in LTBI/COVID-19, we assessed PD-1 and TIM-3 expression to determine whether T CD8+ cells exhibited an exhausted-like phenotype. The frequency of CD8+PD-1+ T cells was higher in LTBI compared to HD in response to stimuli (p<0.05), though no significant difference was observed between COVID-19 and LTBI/COVID-19. Conversely, the frequency of CD8+TIM-3+ T cells was higher in COVID-19 than in LTBI/COVID-19 following stimulation with spike or BCG/spike (p<0.05), although LTBI still displayed a higher frequency compared to HD (p<0.05) (**Figure 5A**).

To confirm cytotoxic function, degranulation capacity was evaluated by CD107a expression. T CD8+ cells from all groups showed similar degranulation capacity following polyclonal stimulation. Degranulation began at 1 hour and peaked at 4 hours post-culture. The frequency of CD8+CD107a+ T cells was higher in LTBI/COVID-19 than in other groups but without statistically significant (**Figure 5B**, left). When evaluating antigen-specific degranulation at 4 hours post-culture, LTBI/COVID-19 exhibited a higher frequency of T CD8+CD107a+ cells compared to COVID-19 when stimulated with BCG (p<0.05), and higher than LTBI when it was stimulated with spike (p<0.05) (**Figure 5B**, right).

Recently, it was highlighted the presence of cytotoxic CD4+ T cells to compensate for exhausted CD8+ T cells in TB patients (27). First, we evaluated the exhausted-like phenotype in CD4+ T cells, and our data showed that the PD-1 expression on CD4+ T cells was unaltered. However, the frequency of CD4+TIM-3+ T cells was elevated in COVID-19 compared to HD at baseline (p<0.01). Under stimulus conditions, LTBI (p<0.01), COVID-19 (p<0.001), and LTBI/COVID-19 (p<0.01) groups exhibited higher TIM-3+ frequencies than HD (**Figure 5C**).

We also observed CD4+ T cells with degranulation capacity (CD107a+) across all groups. The maximum frequency of CD4+CD107a+ T cells at 4 hours post-culture was lower than CD8+CD107a+ T cells (10% vs. 30%, respectively). To note, with polyclonal stimulus, all groups showed a maximum of 10% of CD4+CD107a+ T cells (**Figure 5D**, left); interestingly, under antigen-specific stimuli, the top of degranulation was similar to the observed with polyclonal stimulus, suggesting that most of cytotoxic CD4+ T cells are antigen-specific (**Figure 5D**, right).

### 3.6 The LTBI/COVID-19 coinfection modulates the morpho-functionality of the mitochondria of T cells

**Figure 6:**
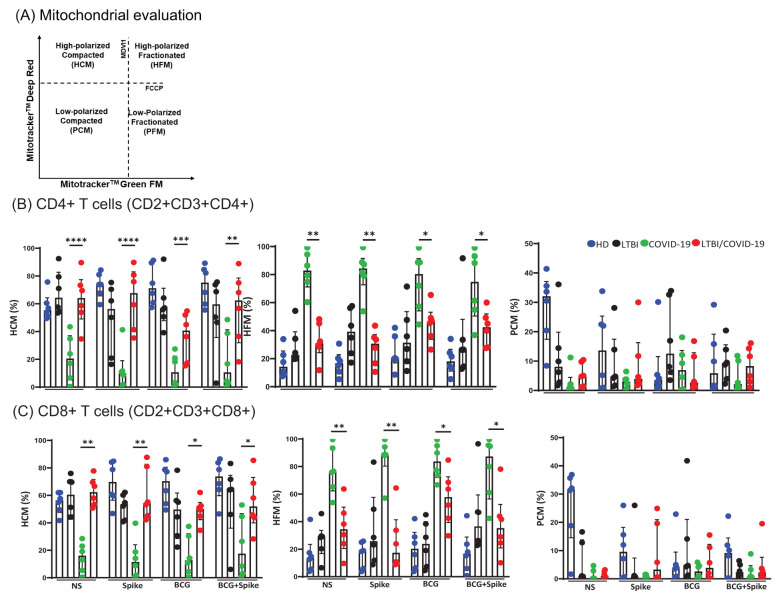
LTBI/COVID-19 coinfection and antigenic stimuli modulate T cells mitochondrial function. Peripheral blood mononuclear cells stimulated with BCG (MOI 1:1) and spike (1μg/mL) were cultured for 24 hours, recovered, and evaluated by flow cytometry (n=6 per group). Into the gate, CD2+CD3+ (T cells), the mitochondrial region selection **(A)**, the frequency of CD4+HCM, CD4+HFM, and CD4+PCM **(B)**, and the frequency of CD8+HCM, CD8+HFM, and CD8+PCM were evaluated. Data are represented as medians with an interquartile range (IQR, 25-75), and each point represents individual data. The statistical comparison was performed using Kruskal-Wallis’s test, *p<0.05, **p<0.01, ***p<0.001, ****p<0.0001. NS, not stimulated; HD, healthy donor; LTBI, latent tuberculosis infection; COVID-19, individual with COVID-19; LTBI/COVID-19, individual with latent tuberculosis and COVID-19 coinfection. HCM, High-polarized compacted mitochondria; HFM, High-polarized fractionated mitochondria; PCM, Low-polarized compacted mitochondria.

The mitochondrial morpho-functionality in T cells was evaluated. Into de CD4+ and CD8+ T cells, the regions evaluated were high-polarized compacted mitochondria (HCM); high-polarized fractionated mitochondria (HFM); low-polarized compacted (PCM) and low-polarized fractionated (PFM) (**Figure 6A**).

The data demonstrated that compared to COVID-19, LTBI/COVID-19 has predominantly CD4+ T cells with high-polarized compacted mitochondria (HCM) at baseline (p<0.0001) and a similar profile is maintained even with stimulus (**Figure 6B**). On the contrary, compared to LTBI/COVID-19, COVID-19 has predominantly CD4+ T cells with high-polarized fractionated mitochondria (HFM) at baseline (p<0.01) and a similar profile is maintained even with stimulus (**Figure 6B**).

A similar behavior was observed in CD8+ T cells, where LTBI/COVID-19 has predominantly HCM CD8+ T cells at baseline (p<0.01) and maintained with stimulus, whereas COVID-19 has predominantly HFM CD8+ T cells at baseline and maintained under stimulus (**Figure 6C**). T cells with low-polarized compacted (PCM) mitochondria were not altered (**Figures 6B, C**).

### 3.7 LTBI/COVID-19 individuals exhibit distinct receptor profiles and unique immunological features compared to COVID-19 individuals

Our results show that LTBI significantly impacts the immune cell profile, and the set of symptoms also differs from those of COVID-19 individuals with or without LTBI. To simplify the molecular immune landscape between groups, a heatmap was made representing the groups evaluated on the vertical axis and the experimental conditions applied for each of them. On the horizontal axis, the molecules and receptors evaluated for the different cell groups are shown. The blue colors indicate lower values, the white colors indicate medium values, and the red colors represent high values.

**Figure 7:**
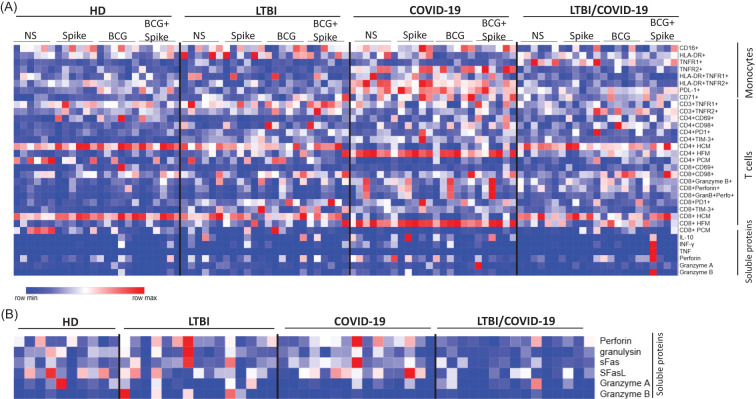
Distinct receptor profiles and immunological features in LTBI/COVID-19 vs. COVID-19 individuals. Heatmap displaying the expression levels of all cell markers evaluated in this study. In this dataset, red indicates increased marker expression, while blue represents decreased expression relative levels. Each column corresponds to one of the six individuals analyzed, categorized by their respective stimulus conditions and classification group. This visualization highlights inter-individual variability and stimulus-specific effects on the immunophenotypic profiles of the evaluated cell populations **(A)**. An evaluation of the plasma soluble proteins was carried out, classifying them according to the group to which they correspond **(B)**, n=10 (HD) and n=15 (LTBI, COVID-19, and LTBI/COVID-19). HD, healthy donor; LTBI, latent tuberculosis infection; COVID-19, individual with COVID-19; LTBI/COVID-19, individual with latent tuberculosis and COVID-19 coinfection, NS, not stimulated. Created in Morpheus, https://software.broadinstitute.org/morpheus.

**Figure 7A** shows that the profile of monocyte subsets from individuals with LTBI/COVID-19 is more similar to HD and LTBI than to COVID-19. Similarly, LTBI/COVID-19 has predominantly CD4+ and CD8+ T cells with highly polarized, compact mitochondria like HD and LTBI, whereas T cells from COVID-19 show highly polarized, fragmented mitochondria (**Figure 7A**). Finally, LTBI/COVID-19 has lower plasmatic levels of cytotoxic molecules compared to COVID-19 (**Figure 7B**), and it is similar to that observed *in vitro* under stimuli (**Figure 7A**).

The data evaluated suggest that the profile of an individual with LTBI/COVID-19 generates a unique immunological signature compared to individuals with COVID-19, which could point to a regulatory role of Mtb in this viral infection, however, further evaluation is needed to determine the phenomenon.

## 4 Discussion

Clinical studies have indicated that COVID-19 can induce the progression of LTBI to active TB in previously infected individuals (6, 8, 28). However, limited studies have highlighted the immunological alterations in LTBI that modulate the immune response during co-infection with COVID-19.

Based on cross-sectional studies, patients with asymptomatic COVID-19 and LTBI showed elevated levels of serum cytokines, chemokines, growth factors, and immunoglobulins compared to patients with COVID-19 alone. Despite not being vaccinated against SARS-CoV-2, these findings suggest a stronger immune response against the virus (18). Similarly, other reports indicated that patients admitted with COVID-19 and LTBI exhibited higher counts and proportions of neutrophils, monocytes, and lymphocytes than those with COVID-19 alone (29).

This study investigates the impact of LTBI in individuals vaccinated against SARS-CoV-2 and with mild COVID-19. Given the nature of our cohort (healthcare workers), all enrolled subjects reported receiving the SARS-CoV-2 and BCG vaccines. It is important to note that the HD control group used to evaluate the phenotypic profile and functional capacity in response to Spike and BCG proteins intentionally included individuals who had previously experienced COVID-19. This choice reflects the current context in the world and allows us to assess immune alterations potentially arising from memory responses generated by prior SARS-CoV-2 infection and/or vaccination, as previously reported (30); in this way, observed differences include the background generated only by the memory. Our data revealed that individuals with COVID-19 displayed a distinct immune profile characterized by a predominance of monocytes expressing pro-inflammatory and regulatory markers, including TNFR2, co-expression of HLA-DR/TNFR2, and CD71. In contrast, the monocyte subsets in LTBI/COVID-19 individuals were more similar to those of healthy donors (HD) and LTBI individuals. Furthermore, compared to COVID-19 individuals, LTBI/COVID-19 individuals showed an increased degranulation capacity of CD8+ T cells, along with similar frequencies of CD8+perforin+ and CD8+Granzime+ T cells, and reduced levels of soluble cytotoxic molecules regardless of the stimuli. Similarly, the levels of soluble cytotoxic molecules in the peripheral circulation were reduced. LTBI/COVID-19 individuals exhibited T cells with highly polarized, compact mitochondria, whereas COVID-19 individuals had highly polarized, fragmented mitochondria.

Previous studies indicated that monocytes from vaccinated mild COVID-19 patients do not show significant alterations (31–33), which is in concordance with our data on HLA-DR+ monocytes. On the other hand, LTBI/COVID-19 exhibited monocyte frequencies more similar than HD, suggesting that LTBI modifies the cell’s presence, probably by a chronic activation. It has been reported that monocytes from LTBI do not modify CD16 and HLA-DR expression after LPS stimulus (34). In our study, we observed a reduction in classical monocytes and an increase in non-classical monocytes in COVID-19, a pattern not observed in LTBI/COVID-19, suggesting a modulatory role of LTBI on monocytes (35). Further research is needed to evaluate their functionality, metabolism, and migration in response to specific antigens to determine whether these differences in monocyte frequencies between COVID-19 and LTBI/COVID-19 aid in better regulation of the inflammatory status.

Numerous studies have associated elevated levels of soluble TNFR1 and TNFR2 with the severity of COVID-19 (25, 36, 37). The COVID-19 individuals enrolled in this study did not present severe or critical illness. Consequently, soluble forms of these receptors were not evaluated. Nevertheless, LTBI/COVID-19 individuals displayed an increased frequency of TNFR1+ monocytes, whereas COVID-19 individuals exhibited TNFR2+ monocytes, primarily within the HLA-DR+ monocyte subset. TNFR1 has been linked to mediating cell death, while TNFR2 is associated with cell survival. Their roles in mycobacterial infections have been extensively studied (38, 39). These results raise new questions, such as whether TNFR1 mediates monocyte subset regulation during LTBI/COVID-19 through cell death promotion, as reported in other contexts (40, 41). On the contrary, TNFR2 may promote monocyte survival, favoring a specific monocyte profile (42).

We did not identify differences in blood parameters, including lymphocyte frequency, between groups. Interestingly, LTBI/COVID-19 individuals exhibited less severe symptoms. Other studies have suggested that LTBI enhances innate and adaptive immunity due to prior Mtb exposure, which may provide a protective effect in COVID-19 co-infection by preventing lymphopenia, a condition associated with higher mortality rates (43). Additionally, we did not observe an altered Th1/Th2 profile, consistent with the findings of Song HW et al. (44).

It is essential to highlight that there are differences between COVID-19 and LTBI/COVID-19, even without stimulation. It is suggested that LTBI/COVID-19 exhibits these immunological changes as a characteristic associated with LTBI, with only minor changes related to stimulation. The Spike protein has been implicated in cytokine release syndrome, favoring severe or critical COVID-19 (45), which was probably not observed in our study because it included only individuals with mild COVID-19. Moreover, all groups reported a mixture of vaccines based primarily on the Spike protein, and previous reports have indicated that immunization influences the response to the Spike protein (11–13). A recent study showed that BCG vaccination does not affect the immune response to COVID-19 or SARS-CoV-2 vaccines such as AstraZeneca or Pfizer (46).

In our study, we cannot determine whether the low response to the Spike protein is influenced by prior BCG vaccination (received in childhood by 94% of the enrolled subjects). It should also be considered that the subjects are healthcare workers with a high risk of exposure to Mtb and other non-tuberculous mycobacteria. This raises a new question about whether exposure to mycobacteria is sufficient to modify the immune response to SARS-CoV-2 antigens.

Unlike CD4+ T cells, CD8+ T cells from LTBI/COVID-19 individuals exhibited increased degranulation capacity upon antigen-specific stimulation. However, levels of soluble cytotoxic molecules were reduced both *in vitro* and *in vivo*. Previous studies have reported that COVID-19 patients experience diminished cytotoxic responses, with CD4+ T cells acquiring a cytotoxic profile to compensate for CD8+ T cell dysfunction (47). It has also been suggested that CD8+ T cells with low perforin levels during the acute phase of severe SARS-CoV-2 infection may predict long COVID (48). Moreover, decreased levels of sFas and sFasL have been linked to compromised clearance of SARS-CoV-2-infected cells, adverse outcomes, and increased risk of organ failure (49, 50).

Previous studies have demonstrated that exhaustion markers, such as CD39 and TIM-3, are elevated on CD8+ T cells during COVID-19 and active TB, while PD-1 is not prominently expressed (27, 51–53). However, in our study, we did not observe an exhausted profile of T cells. Although the expression of the regulatory molecule TIM-3 was altered on both CD4+ and CD8+ T cells, this alone does not indicate an exhausted profile. Instead, it may be associated with the discrepancy between intracellular and soluble cytotoxic molecule levels observed in LTBI/COVID-19 individuals.

Regarding the increased degranulation capacity of CD8+ T cells in LTBI/COVID-19 individuals, it has been previously reported that CD8+ T cells from individuals living with HIV and with an undetectable viral load exhibit a potent cytotoxic response following SARS-CoV-2 vaccination (54). Given the low levels of soluble cytotoxic molecules, an important question arises: How do these cells effectively control mycobacterial growth or viral replication? We speculate that, although cells from LTBI/COVID-19 individuals display efficient degranulation in response to stimuli, their cytotoxic capacity may not be optimal, as suggested by the low levels of cytotoxic molecules. This finding aligns with the impaired cytotoxic function observed in TB patients, likely due to chronic antigen exposure (27), which could potentially compromise pathogen control. Alternatively, it might represent a regulatory mechanism to prevent excessive immune activation and tissue damage. However, further studies are needed to respond to this speculation and determine the true consequences of this observation.

Although the presence of CD4+ T cells with a cytotoxic phenotype has been reported in both COVID-19 and TB (27, 48), our study specifically identified that LTBI/COVID-19 individuals possess these unconventional CD4+ T cells, which appear to be entirely antigen-specific.

Mitochondrial fragmentation has been associated with altered metabolism, characterized by decreased cellular energy levels, increased apoptosis, excessive production of toxic molecules, and reduced immune response efficacy (10, 55). Our data demonstrated that LTBI/COVID-19 individuals predominantly exhibit T cells with a mitochondrial profile distinct from that observed in COVID-19 individuals. These findings suggest that SARS-CoV-2 induces mitochondrial stress, consistent with several studies highlighting this process in viral infections (55–57).

However, pre-existing chronic infections such as LTBI appear to condition a different mitochondrial response, which could be crucial for maintaining diverse cellular mechanisms. In the context of LTBI/COVID-19, lower mitochondrial fragmentation does not necessarily indicate cellular well-being; instead, it may reflect a more balanced state of mitochondrial dynamics, potentially influenced by LTBI. A study on acute COVID-19 demonstrated that increased mitochondrial mass could prevent apoptosis, creating an intracellular environment favorable for virus propagation in infected cells (10, 55).

Further research, including specific metabolic investigations, is needed to determine whether this phenomenon is driven by mitochondrial fusion or fission mechanisms. Additionally, these studies may aim to elucidate the potential long-term immunometabolic consequences in the affected individuals.

Our study is not free from limitations. First, we cannot clarify the timing of the last COVID-19 diagnosis in the reference groups (HD and LTBI). Second, we are unable to determine if there is any immunological involvement of the BCG vaccine and prolonged exposure to Mtb respiratory disease in the recruited individuals. Finally, we cannot ascertain with QuantiFERON how long the patient was infected with Mtb, thus it is also not possible to determine whether individuals with LTBI still harbor viable bacilli that could induce subsequent Mtb development or if these bacilli trigger ongoing immunologic changes.

## 5 Conclusion

The findings reveal significant alterations in monocytes and T cells of individuals with LTBI/COVID-19, suggesting that coinfection of both pathologies may induce changes in the immune phenotype, impacting cytotoxic function and cellular activation. However, further investigation is necessary to elucidate the mechanisms underlying these responses and to ascertain their impact on individuals’ clinical progression.

## Data Availability

The original contributions presented in the study are included in the article/**Supplementary Material**. Further inquiries can be directed to the corresponding author.
