# Supplementary material for: Latent tuberculosis coinfection in mild COVID-19 is associated with a distinct immune cell phenotype marked by enhanced cytotoxic degranulation and mitochondrial alterations
Source: Front Immunol. 2025 May 19;16:1566449. doi: 10.3389/fimmu.2025.1566449 (PMC12127376; doi:10.3389/fimmu.2025.1566449)
Supplement: Supplementary file 1 [file DataSheet1.pdf]

Supplementary material

1 Statistical analysis (sample size)

The sample size of the recruited subjects was determined based on convenience sampling. However, post-hoc power analyses demonstrated that the study was adequately powered to detect the observed effects. Specifically, the power calculations yielded values of 80% for degranulation and 89% and 90% for cell phenotype analyses. These values reflect a high probability of detecting true effects, thereby supporting the robustness and reliability of the findings reported in this study. The post-hoc "Power analysis for two proportions" was calculated as follow, considering the groups' COVID-19 and LTBI/COVID-19.

Degranulation

$$\text{Power} = \Phi\left\{ -Z_{1-\alpha/2} + \frac{\Delta}{\sqrt{\sigma_1^2/n_1 + \sigma_2^2/n_2}} \right\}$$

$$\text{Power} = \Phi\left\{ -(1.96) + \frac{7.66}{\sqrt{\ 1.67^2/4 + 5.204^2/4}} \right\}$$

$$\text{Power} = \Phi\left\{ 0.843 \right\} = 0.8 = 80\%\ \text{power}$$

Cell phenotype

Monocytes

$$\text{Power} = \Phi\left\{ -Z_{1-\alpha/2} + \frac{\Delta}{\sqrt{\sigma_1^2/n_1 + \sigma_2^2/n_2}} \right\}$$

$$\text{Power} = \Phi\left\{ -(1.96) + \frac{9.605}{\sqrt{\ 1.684^2/6 + 7.177^2/6}} \right\}$$

$$\text{Power} = \Phi\left\{ 1.232 \right\} = 0.891 = 89.1\%\ \text{power}$$

T cells

$$\text{Power} = \Phi\left\{ -Z_{1-\alpha/2} + \frac{\Delta}{\sqrt{\sigma_1^2/n_1 + \sigma_2^2/n_2}} \right\}$$

$$\text{Power} = \Phi\left\{ -(1.96) + \frac{51.28}{\sqrt{\ 22.08^2/6 + 14.9^2/6}} \right\}$$

$$\text{Power} = \Phi\left\{ 2.756 \right\} = 0.997 = 99.7\%\ \text{power}$$

n_1 = sample size for group #1

n_2 = sample size for group #2

$\Delta = |\mu_2-\mu_1|$ = absolute difference between two means

σ_1, σ_2 = variance of mean #1 and #2

α = probability of type I error (usually 0.05)

β = probability of type II error (usually 0.2)

Z = critical Z value for a given α

$\Phi\{\}$ = function converting a critical Z value to power

2 Supplementary tables

Table S1. Antibodies used for flow cytometry

Antibody	Conjugate	Clone	Catalog	Company
**Used in extracellular staining**				
CD2	PE	PA2.10	300208	Biolegend
CD3	BV510	UCHT1	300448	Biolegend
CD3	APC-Cy7	HIT3a	300318	Biolegend
CD14	PerCP CY5.5	HCD14	325622	Biolegend
CD16	APC-Cy7	B73.1	360710	Biolegend
HLA-DR	AF700	L243	307625	Biolegend
CD4	PE	SK3	980804	Biolegend
CD4	APC-Cy7	RPA-T4	250049T100	TONBO Bioscience
CD4	PerCP	RPA-T4	347324	BD Bioscience
CD8	PE-Cy7	SK1	344750	Biolegend
CD8	PACIFIC BLUE	HIT8a	300928	Biolegend
CD8	FITC	RPAT8	301050	Biolegend
CD69	CD605	FN550	562989	BD Bioscience
CD71	BV650	C4164	334115	Biolegend
CD98	BV786	UM7F8	744504	BD Bioscience
CD107a	APC	W18263B	301104	Biolegend
CD120a	APC	W15099A	369906	Biolegend
CD120b	PE-Cy7	367A02	358412	Biolegend
CD274	BV711	29E.2A3	329722	Biolegend
CD279	BV650	NAT105	367430	Biolegend
TIM-3	BV605	F38-2E2	345017	Biolegend
**Used in intracelular satining**				
T-BET	PE-Cy7	4B10	644824	Biolegend
GATA-3	BV421	16E10A23	653814	Biolegend
GRANZYME B	AF700	QA16A02	372222	Biolegend
PERFORIN	APC	B-D48	353312	Biolegend
**Others**				
MitoTracker Green FM	FITC	NA	M22426	ThermoFisher
MitoTracker Deep Red	APC	NA	M7514	ThermoFisher
Fixable Viability Dye	PE-Texas Red	NA	423109	Biolegend
Legendplex Human CD8/NK panel (13 plex)	NA	NA	741187	Biolegend

NA, not applicable

Table S2. Demographic data of study groups

	HD<br>n=20	LTBI<br>n=15	COVID-19<br>n=52	LTBI/COVID-19<br>n=15
**Demographic Data***				
Age (years)	34(10)	35(5)	35 (10)	37 (9)
**Sex****				
Male	5 (25)	4 (27)	20 (38)	4 (27)
Female	15(75)	11(73)	32(62)	11(73)
**Comorbidities****				
Hypertension	0	1 (7)	4 (8)	2 (14)
Diabetes	1 (5)	0	4 (8)	0
Overweight	2(10)	1 (7)	22(42)	3(20)
Obesity	0	1(7)	20 (38)	4(27)
**SARS-CoV-2 vaccines****				
Two doses	3(15)	2 (13)	6(12)	2(14)
Three doses	13(65)	6(40)	35(67)	8(53)
Four doses	3(15)	7(47)	8(16)	5(33)
Five doses	1(5)	0	3(5)	0
**COVID-19 (times the disease was diagnosed)****				
Never	5(25)	2 (13)	0	0
Once	5(25)	5(33)	6(12)	0
Twice	3(15)	6(40)	25(47)	7(47)
Three	5(25)	1(7)	16(31)	6(40)
Four	2(10)	1(7)	5(10)	2(13)
**Contact with TB patients****	10(50)	9(60)	21(40)	7(47)

Data showed: *mean (%), **n (%)

Table S3. Laboratory features

	HD n=20	LTBI n=15	COVID-19 n=52	LTBI/COVID-19 n=15	P value
**Blood routine parameters***					
Leukocytes (4.9-10.9 x 10^3/mm^3)	6.4 (4.3-8.8)	7.3 (4.2-9.9)	6.1 (1.9-10.8)	6.4 (4-14)	ns
Lymphocytes (21-48%)	34 (17-54)	30 (29-40)	33 (13-58)	31 (9-53)	ns
Neutrophils (39-68%)	56 (35-70)	61 (49-74)	54 (31-77)	55 (39-84)	ns
Monocytes (4-10%)	7.3 (3.2-13.1)	6.5 (4.4-8.7)	7.2 (3.5-14.2)	6.7 (4.5-10.2)	ns
Platelets (175-388 10^3/mm^3)	286 (214-410)	317 (254-430)	270 (173-422)	307 (232-566)	ns
Glucose (70-99)	90 (70-161)	89 (74-100)	92 (70-172)	90 (75-102)	ns
**Glycated hemoglobin (HbA1c)****					
< 5.7 % normal	13 (65)	10 (67)	34 (65)	10 (67)	--
5.7-6.4% prediabetic	6(30)	5 (33)	14 (27)	5 (33)	--
6.5% diabetic	1(5)	0	4 (8)	0	--

The data are shown as *mean (min, max), **n (%), and ns= not significant

3 Supplementary Figures

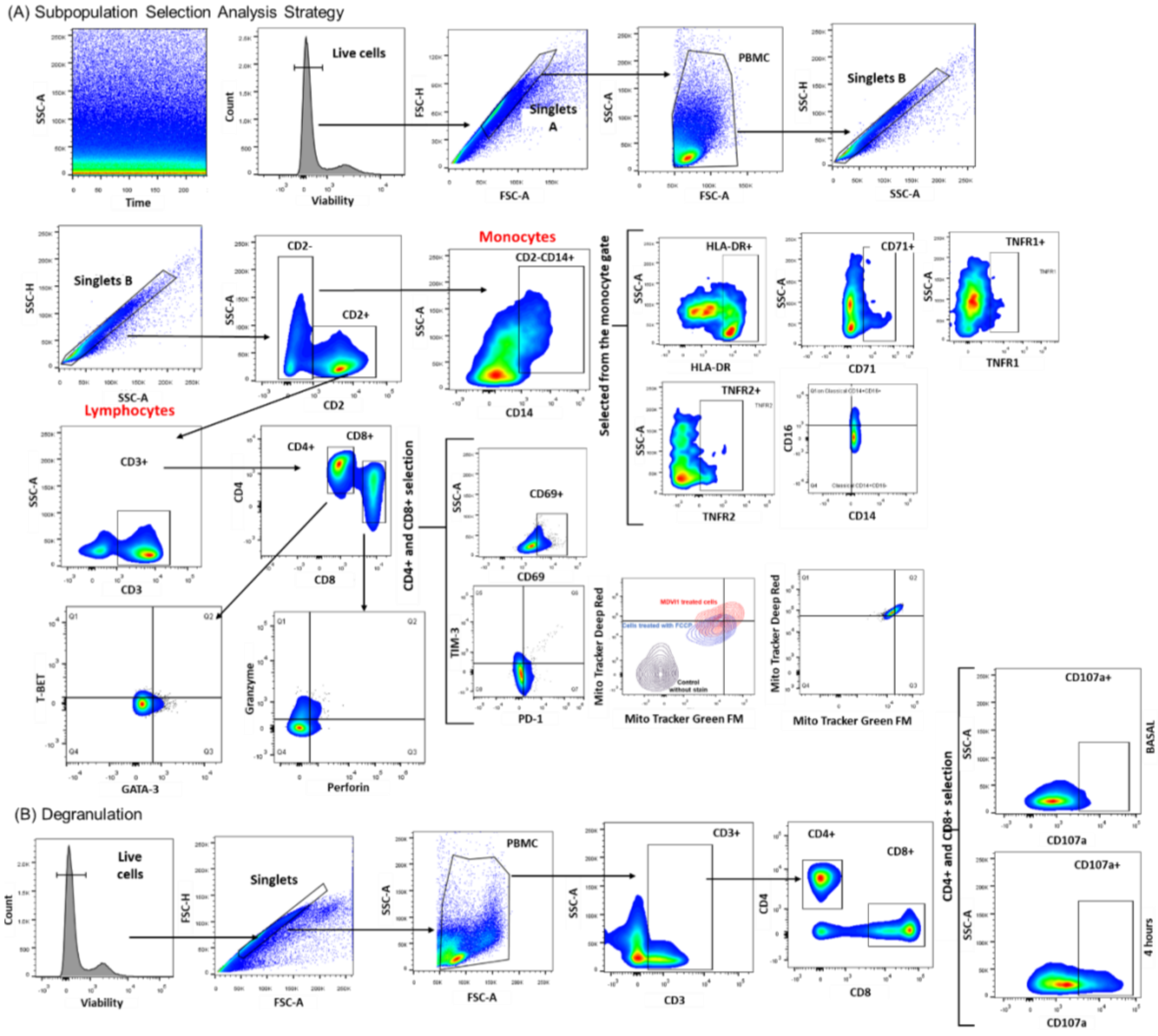

Figure S1. This flow cytometry strategy characterized monocyte and T cell populations from peripheral blood mononuclear cells (PBMC) of the four groups included in the study. **A)** Plotting data versus time was performed to identify problems and remove questionable events. Viable cells were selected, using singlet analysis which employed forward scatter (FSC-A vs. FSC-H), PBMC were selected and after performing a second singlet based on (SSC-A vs. SSC-H) as parameters, additional analyses of CD2- or CD2+ cells, monocytes (CD2-CD14+), and T cells (CD2+CD3+), along with their CD4+ or CD8+ subpopulations. For monocytes, activation markers and death or survival receptors were evaluated within the CD14+ gate, including HLA-DR, CD71+, classical monocytes CD14+CD16-, non-classical monocytes CD14+CD16+, TNFR1, and TNFR2+. In the T cell gate, activation and regulatory markers (CD69+, PD-1, and TIM-3) were assessed. Additionally, in the CD4+ gate, GATA-3 and T-BET were evaluated, while in the CD8+ gate, granzyme and perforin were analyzed. Conversely, mitochondrial evaluation classified cells into CD4+ and CD8+ subpopulations, which were further subdivided based on mitochondrial mass (using

MitoTracker Green FM) and mitochondrial polarization (using MitoTracker Deep Red). **B)** For degranulation, viable cells were selected, using singlet analysis employing forward scatter (FSC-A vs. FSC-H), subsequently using gate (FSC-A vs. SSC-A) PBMC were selected. Subsequently, CD3+ cells were selected to subsequently obtain the gate of CD4+ and CD8+ T cells; within these cells, either CD4+ or CD8+ CD107a was evaluated to determine the cytotoxic capacity in the established times, as an example, the basal time and the four-hour time are presented.

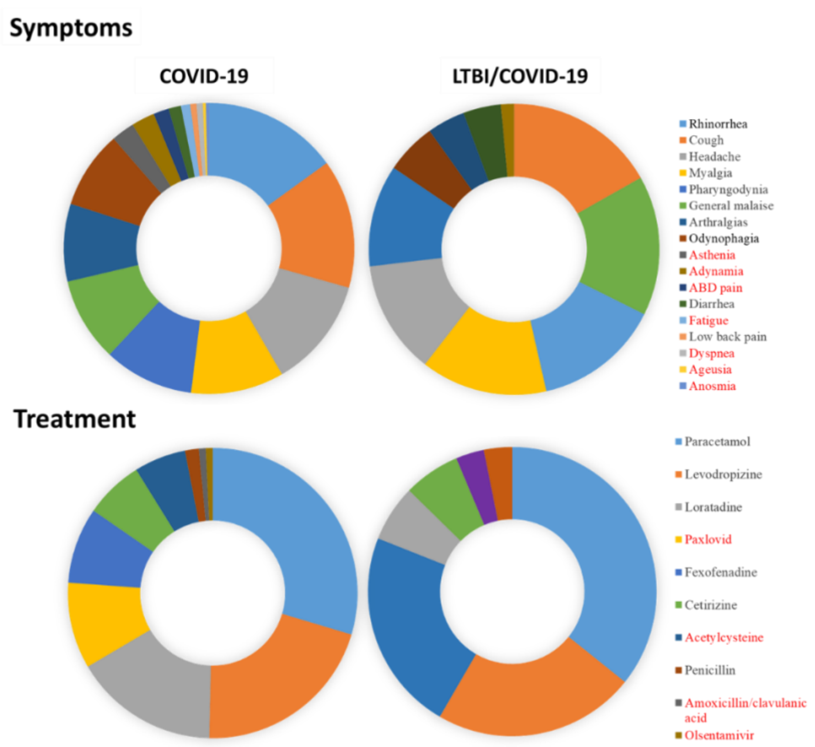

Figure S2. Prevalence of Symptoms and Pharmacological Treatments in Patients with COVID-19 and LTBI/COVID-19. The donut charts illustrate the distribution of symptoms and treatments in each group. Symptoms and treatments indicated in red were present in the COVID-19 group but not in LTBI/COVID-19. ABD: abdominal pain.

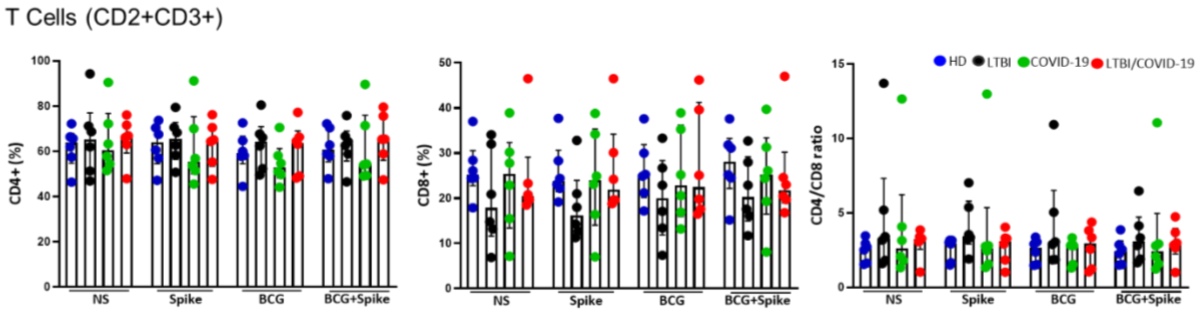

Figure S3. The frequency of T cells is similar in all groups tested. Peripheral blood mononuclear cells stimulated with BCG (MOI 1:1) and spike (1µg/mL) were cultured for 24 hours, recovered, and evaluated by flow cytometry. Into the gate CD2+CD3+ (T-cells), the frequency of CD4+ and CD8+ T cells was evaluated. Data are represented as medians with an interquartile range (IQR, 25-75), and each point represents individual data. The statistical comparison was performed using Kruskal-Wallis's test. NS: not stimulated. HD: healthy donor; LTBI: latent tuberculosis infection; COVID-19: individual with COVID-19; LTBI/COVID-19: individual with latent tuberculosis and COVID-19 coinfection.

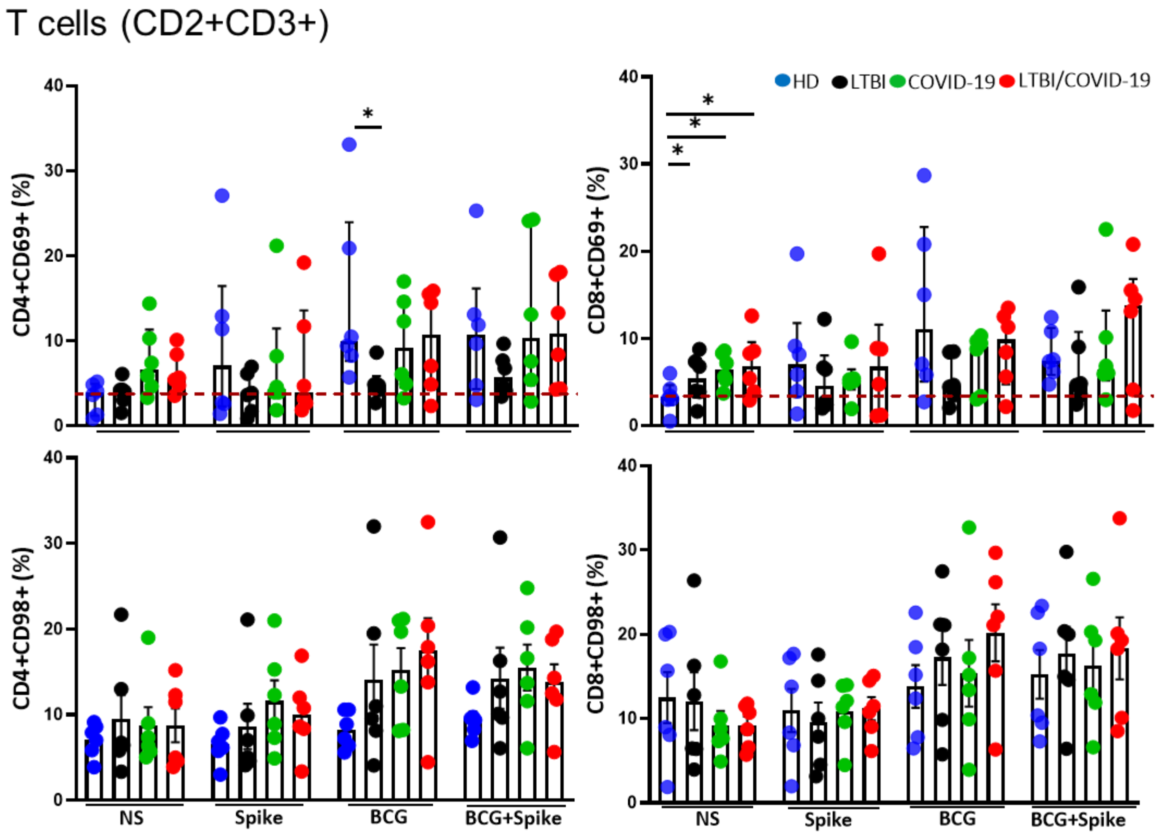

Figure S4. Activation markers show no differences in the LTBI/COVID-19 vs COVID-19. Peripheral blood mononuclear cells stimulated with BCG (MOI 1:1) and spike (1µg/mL) were cultured for 24 hours, recovered, and evaluated by flow cytometry. Into the gate CD2+CD3+ (T cells), the frequency of CD4 and CD8 T cells expressing CD69+ and CD98+, were evaluated. Each point represents the monocyte frequency of six independently evaluated individuals. The dotted red line represents the median of the HD not stimulated. Data are represented as medians with an interquartile range (IQR, 25-75), and each point represents individual data. The statistical comparison was performed using Kruskal-Wallis's test. NS: not stimulated. HD: healthy donor; LTBI: latent tuberculosis infection; COVID-19: individual with COVID-19; LTBI/COVID-19: individual with latent tuberculosis and COVID-19 coinfection.

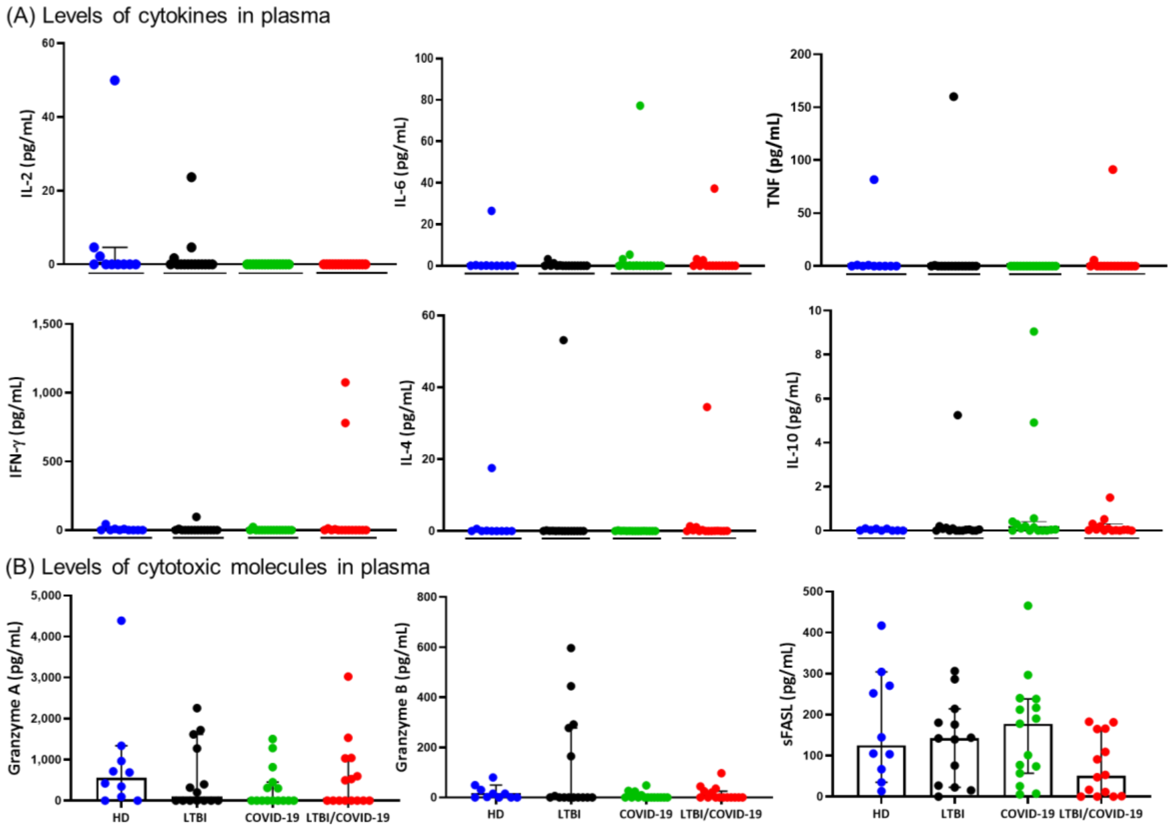

Figure S5. Plasma cytokines are in functional balance and some cytotoxic molecules. Evaluation of the proinflammatory and anti-inflammatory cytokines concentration for CD4+ T Cells **(A)**. Evaluation of cytotoxic molecules **(B)** were evaluated by LEGENDplex assay, n=10 (HD) and n=15 (LTBI, COVID-19, and LTBI/COVID-19). Data are median with interquartile range (IQR, 25-75). Statistical comparison was performed using the Kruskal-Wallis test. HD: healthy donor; LTBI: latent tuberculosis infection; COVID-19: individual with COVID-19; LTBI/COVID-19: individual with latent tuberculosis and COVID-19 coinfection.
